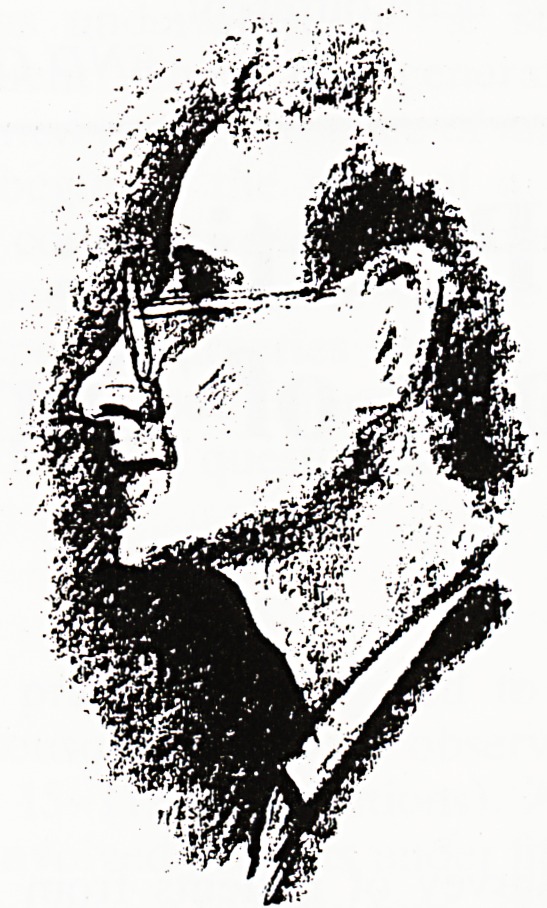# Eating Tomorrow

**Published:** 1988-11

**Authors:** K. W. Heaton

**Affiliations:** University Department of Medicine, Bristol


### Eating Tomorrow

**Dr K. W. Heaton**
University Department of Medicine, Bristol

Dr Heaton began by saying that he would not dare to forecast future eating habits, those who have done so in the past have been shown to be rather foolish. Ten years ago a food industry spokesman stated that "the food of the future will be lurid in colour and taste, quickly prepared, quickly eaten and quickly forgotten". It is true that the listed ingredients in packaged foods sometimes seem to to consist mainly of synthetic substitutes, artificial flavourings and preservatives. There are 50 different artificial colours available to British food manufacturers and 17 of these are totally synthetic. However, a recent survey in *The Times* had shown that half the housewives in Britain are actually making alterations in their food consumption because of health concerns. The foods losing sales as a result are dairy products, salt, meat, sugar, and products with additives and preservatives. He only felt confident to predict that tomorrow's food would be different from today's. He preferred to talk about changing trends.

Looking back into the past records of the BRI 250 years ago he found details of what they were eating. For breakfast a pint of broth or milk pottage, for dinner 10 ozs of beef or mutton for 4 days a week, on the other 3 days a pint of rice milk or 'pap'. For supper a pint of broth or 2 ozs of cheese alternately. 12 ozs of bread and 3 pints of small beer were given daily to each patient. This diet contains very little Vitamin C or Folic acid. This was the diet of working people. The rich fared rather better. Parson Woodforde's diary gives a famous account of a dinner party he gave on April 20th 1774. They had cod, mutton, soup, chicken pie, pigeons and asparagus, fillet of veal with mushrooms and high sauce, roasted sweetbreads, hot lobster, apricot tart and 'in the middle a pyramid of syllabubs and jellies', 'after dinner we had dessert of fruit, madeira, white and red port'. In 1737 very tiny amounts of sugar were eaten in comparison with today, sugar was a luxury enjoyed only by the gentry and the rich. Bread was the staple food of the common people who ate a pound a day on average. Since then bread consumption has fallen and sugar consumption has risen. Since 1880 grain production has dropped drastically, and the consumption of meat, sugar, vegetables and fruit, dairy products, fats and oils which are more expensive and also tastier has increased. During the last 15 years however eating habits have changed more rapidly and more drastically than at any previous time in history. Since 1975 after 200 years of decline, bread eating has stablised, much to the relief of the millers and bakers. Though white bread consumption has continued its decline, wholemeal bread eating has risen increasingly steeply, an entirely new phenomenon. In supermarkets frequented by the 'educated classes' 50% of the bread now being sold is brown. 10 years ago it was under 20%.

An institution unique to this country is the National Food Survey started by the Ministry of Agriculture, Fisheries and Food in 1940. It surveys 7-8,000 representative households, in which every item of food brought into the home is recorded and analysed. It has revealed that since 1975 we are drinking less milk as a nation, and we are drinking skimmed rather than whole milk, the only possible reason for this is that we have got the wind up about dairy fat – a health oriented change. There is also a dramatic decline in butter consumption, replaced by vegetable oils and margarine. Also, a steady decline in sugar consumption as packet sugar, though the consumption of biscuits, cakes and preserves which also contain sugar, is not declining at all. Consumption of confectionery, especially chocolate confectionery is actually rising quite steadily. This he regards as a minor triumph for hedonism. Another advance of hedonism is the increasing consumption of wine, cider and perry. There is a rapid increase in the consumption of frozen meat, no doubt due to the convenience of keeping a stock in the freezer at home. The British breakfast is changing, bacon and eggs are going down while cereals and fruit juice are going up, partly no doubt due to convenience but also due to a healthy desire to reduce fat and cholesterol.

The new factor in deciding eating habits is health. The media have a lot to do with this, especially the women's magazines which are full of articles about healthy eating. The supermarkets are also promoting healthy eating by displaying leaflets with nutrition information. The housewife is now becoming sophisticated in her nutrition knowledge and obliges doctors also to keep abreast of developments.

A revolutionary range of new foods is now being developed. Based upon 'mycoproteins', these are a synthetic product made from a garden soil fungus, Fusarium Graminearum, which is processed and flavoured to resemble chicken, veal, beef or lamb. From this a whole range of savoury pies has been produced, under the emotive name 'Quorn', when cooked they look nice and taste good. 70 British Home Stores restaurants throughout the country sell them, also made up as goulash soup or with rice. It is promoted as a health food because unlike meat it is low in fat and cholesterol and rich in fibre and, like meat, rich in protein. It is selling extremely well.

The Royal College of Physicians has led the way with reports on 'Diet and Coronary Disease', on 'Medical Aspects of Dietary Fibre' and on 'Obesity'. The DHSS has also produced a booklet on 'Eating for Health'. The Committee On Medical Aspects of Food Policy produced an important document 'Diet and Cardiovascular Disease' and the British Nutrition Foundation has recently produced a report on 'Sugars and Syrups'. The most influential report was the 1983 NACNE Report 'Nutritional Guidelines for Health Education in Britain'. This became famous because the Government disowned it and the media became suspicious. It was the first official document to make recommendations about eating habits in terms of actual amounts of food we should be eating and the changes we should make in our diet. The Royal Society of Medicine three years ago established a forum on food and health consisting now of nearly 300 members. Since then there have been 11 meetings including one on district food and health policies. Bristol Health District has such a policy as do nearly all other districts. A consensus has been emerging that Western diets have too many calories, too much saturated fat, too little fibre and that they are cariogenic. Diseases that are being talked about as being caused by diet are not only cardiovascular, strokes and heart disease, but also cancers. In 1981 Doll and Peto in a report commissioned by the United States suggested that the incidence of many cancers was influenced by diet, including cancers of the stomach and large bowel, the body of the uterus, gall bladder and in tropical countries of the liver, possibly also of the breast and pancreas. Practicable dietary means might possibly reduce the incidence of these cancers by as much as 35%. Fortunately the type of diet likely to prevent cancer is the same as that thought to prevent coronary heart disease, gallstones, maturity onset diabetes, gout and many other diseases of the Western World. However there is room for scepticism and need for more research.

The French, the world experts on eating and drinking, ought to be given the last word. Brillat-Savarin, writing 10 years after the Battle of Waterloo, said that 'the destiny of nations depends on what they eat'. An example might be that if Napoleon had eaten more fibre he would not have been suffering from an attack of piles during the battle and the outcome might have been different!

*END OF SYMPOSIUM*

## Figures and Tables

**Figure f1:**